# The Effect of Social Exclusion on Trust Among Youth Orphaned by HIV/AIDS: Evidence From an Event-Related Potentials Study

**DOI:** 10.3389/fpsyt.2022.898535

**Published:** 2022-07-14

**Authors:** Jiaojiao Wan, Qi Zhao, Yafei Zhang, Lili Ji, Junfeng Zhao, Shan Qiao, Xiaoming Li

**Affiliations:** ^1^School of Psychology, Institute of Behavior and Psychology, Henan University, Kaifeng, China; ^2^Department of Psychology, Faculty of Social Sciences, University of Macau, Macao, Macao SAR, China; ^3^Department of Health Promotion, Education, and Behavior, University of South Carolina, Columbia, SC, United States

**Keywords:** social exclusion, trust, youth orphaned by HIV/AIDS, ERP, FRN, P300

## Abstract

Grounded in a follow-up study among children who lost one or both parents to HIV in central China in the early 2000s, we conducted an event-related potentials (ERPs) experiment to explore the effect of social exclusion on trust and the corresponding neurophysiological mechanism among youth orphaned by HIV/AIDS (“AIDS orphans”). A sample of 31 AIDS orphans (26.16 ± 3.34 years old; 15 female) and 32 age and development status matched controls (25.02 ± 3.45 years old; 14 female) participated in the study. They were all assigned to play Cyberball, a virtual ball-tossing game that reliably induced social exclusion (15 orphans, 16 controls) and inclusion (16 orphans, 16 controls). Then, they played the Trust Game by taking the role of trustor with their electroencephalograms (EEGs) being recorded during the game. In the Trust Game, each participant was required to decide whether to trust their partners in over 150 trials (decision-making stage). The partner’s reciprocation strategies were pre-programmed by the experimenter (with an overall reciprocating rate of 50%). All participants were provided with post-decision feedback about the outcome of their decisions (gain or loss of game points) in each trial (outcome evaluation stage). We analyzed their behavioral responses at the decision-making stage and ERP components at the outcome evaluation stage. Behavioral results showed that the proportion of orphans choosing trust was significantly higher than the controls, and the trust ratio of the orphan exclusion (OE) group was significantly higher than that of the orphan inclusion (OI) group, control exclusion (CE) group, and control inclusion (CI) group. Furthermore, the response time of the OE group was significantly shorter than that of other groups. ERP results indicated that the amplitude of the feedback-related negativity (FRN) in the OI group was significantly more negative than that in the CI group with loss feedback, while there was no significant difference between the OE and OI groups. Similarly, the P300 amplitudes following outcome feedback were larger in the CI group than that in the OI group with gain feedback and had no significant difference between OE and OI.

## Introduction

In the late 1980s, many rural residents were infected with HIV in Henan province in China, an agricultural province with a population of 96.66 million, because of the unhygienic blood collection (1). Although the commercial blood/plasma collection has been banned by the Chinese government since 1998, the infection has spread widely among former commercial blood/plasma donors and their spouses. The average HIV prevalence rate in this population was 10–20% and even exceeded 60% in some communities, and there were at least 100,000 AIDS orphans in China by 2004. In the year 2020, about 1.053 million people were living with HIV, and 351,000 cumulative reported deaths in China (2). Meanwhile, globally, there were 690,000 adults and children’s deaths, aged 0–17 years old, and 240,000 orphans’ deaths due to AIDS (3). Children who were orphaned due to the death of parents infected with HIV/AIDS faced many challenges, including parental death, disruption of schooling, stigma, social exclusion, and other negative psychological impacts (4–6). All these early negative events can be extremely stressful for youth orphaned by HIV/AIDS (orphaned youth) and significantly affect their social and interpersonal adaptation during childhood and young adulthood.

Trust is the foundation of interpersonal communication and is a paradoxical phenomenon that encompasses both lofty aspirations and deep fears (7). For example, an online shopper whose product, although already paid for, is not delivered. In other words, the trustor must predict whether the other person is trustworthy. As a result, (8) defined trust as “a psychological state comprising the intention to accept vulnerability based upon positive expectations of the intentions or behavior of another”. Based on the above definition, some common characteristics should be included in the laboratory measurements of trust: (a) the trustor has two choices of trust or distrust; (b) choosing trust has potential benefits, but, at the same time, it needs to face risks and vulnerabilities; (c) the outcome of trust depends on the behavior of trustee. The Trust Game is the most used game paradigm among the laboratory measurements of trust (9, 10).

In the Trust Game, the trust decision-making process includes the behavioral decision stage (the trustor chooses to trust or distrust) and the outcome evaluation stage (after choosing, the trustor evaluates the outcome resulting from the trustee’s behavior). As trust and distrust behavioral decisions are woven in with the complex social environments addressed in the Trust Game (11), exploring how outcomes are evaluated provides the opportunity to understand how decisions are made. In addition, people often rely on the effective coding and processing of previous results to adjust subsequent behavioral strategies and choices, and then make more appropriate and quick choices (12). Therefore, focusing on the outcome evaluation stage is also one of the most important indicators reflecting trust performance. Psychophysiological research purported that the brain had developed special mechanisms to quickly assess the valence and magnitude of outcomes, as well as their subjective and motivational significance (13). Researchers observed two ERP components related to the outcome evaluation: the feedback-related negativity (FRN) and positive electrophysiological potential P300 (14, 15). FRN is a negative potential at fronto-central recording sites and peaks around 250–300 ms after stimulus onset. Moreover, loss feedback induces more negative FRN than gain feedback (16). Some researchers believed that FRN reflects the individual evaluation of the emotional motivation, meaning, of feedback stimulus (14), while other researchers believed that FRN reflects the degree of deviation between feedback results and prior expectations (expectancy-deviation), and FRN was greater when observing the larger expectancy-deviation (17, 18). P300 is a positive potential and peaks around 300–600 ms after stimulus onset. Researchers believed that P300 was sensitive to the number of feedback results (19, 20) and might reflect the process of attention resource allocation and relevant social information in outcome evaluation (13, 15).

It can be seen from the above that the behavioral decision (trust vs. distrust) and the EEG indicators (FRN and P300) related to the outcome evaluation in Trust Game can reflect the performance of trust. However, the important issues we are concerned with are what are the factors and how to affect trust, especially for orphaned youth?

Social exclusion is one of the most likely challenges faced by orphaned youth in growth and is also a possible negative factor for orphaned youth in interpersonal trust. Studies have shown that the higher the experience of social exclusion, the lower the trust toward others (21). DeWall et al. (22) found that individuals would generate the hostile cognition toward the rejector and irrelevant others after being temporarily rejected by strangers in the laboratory and reduce trust toward the rejector in the subsequent Trust Game. A previous neuroimaging study of social exclusion suggested that people with high rejection sensitivity showed less activation of the right ventrolateral prefrontal cortex (rVLPFC), while viewing representational paintings depicting themes of interpersonal interaction (23), and rVLPFC is positively correlated with trust (24). Similarly, we inferred that orphaned youth might show less trust when they encountered social exclusion.

However, how does social exclusion affect the trust of orphaned youth? In another word, what is the feature of trust decision-making process (behavioral decision stage and outcome evaluation stage) in the Trust Game after orphaned youth experienced social exclusion? In the laboratory, social exclusion can be induced by the Cyberball game, which has proven to be a reliable paradigm to elicit exclusion-related distress (25). In Cyberball, subjects can be divided into social exclusion condition and social inclusion conditions (control conditions). After being ostensibly excluded by two peers in Cyberball, subjects consistently reported heightened levels of distress in the form of higher levels of negative mood, and lower sense of belonging, control, and self-esteem (26, 27). Therefore, compared with the social exclusion condition, orphaned youth in the inclusion condition may expect more reciprocal results from each other. Therefore, the inclusion condition may trigger higher expectations about the outcome of the game than the exclusion condition. Based on previous studies, we predicted that the FRN amplitude would be larger in the inclusion condition with loss feedback. In addition, according to the existing empirical results of P300, the results of win-win cooperation represent not only the material rewards, but also the social meanings. Thus, we expected that the gain feedback might trigger a larger P300 in the inclusion condition than that in exclusion condition.

Previous studies on orphaned youth have mostly focused on issues, such as resilience and mental health intervention after negative experience, and have paid little attention to interpersonal adaptation (28, 29), such as trust. Especially, little is known about the situation of trust when orphaned youth encountered social exclusion. Accordingly, the current study aimed to explore the effect of social exclusion on trust and the corresponding neurophysiological mechanism among youth orphaned by HIV/AIDS. Therefore, we used Cyberball and Trust Game to induce social exclusion and trust, combined with ERP research, aimed to examine the behavioral decision and EEG indicators of outcome evaluation in orphaned youth when social exclusion was encountered. Specifically, we propose two hypotheses: first, we hypothesized that the trust rate of exclusion condition would be lower than the inclusion condition. Second, we hypothesized that the FRN amplitude would be larger in the inclusion condition with loss feedback than that in the exclusion condition, and the P300 amplitude would be larger in the inclusion condition with gain feedback than that in the exclusion condition.

## Materials and Methods

### Participants

The participants were from a larger sample of a psychological assessment study in central China about 15 years ago, which has been described in detail elsewhere (30). Briefly, the orphan sample was recruited from four government-funded orphanages and eight small group homes. We worked with the village leaders to generate lists of families caring for orphans by HIV/AIDS, approached the families on the lists, and recruited one child per family to participate in the assessment. When there were siblings in an orphanage, group home, or household, a single child was randomly selected. The control sample was recruited from the same villages where the orphans were recruited. We worked with the village leaders to create a list consisting of households, in which no one was known to be HIV-infected or died of HIV/AIDS. At this time, we re-contacted 64 participants (orphaned youth and controls) in the prior study through local schools and invited them to participate in the current study. As shown in Table 1, the participants in the current study consisted of 34 males (53.12%) and 30 females (46.88%). The average age was 25.79 years. In the case of orphaned youth, almost ninety-four percent of the orphans considered themselves as having “very good” or “good” health. Most orphans reported their monthly income was under ¥6,000 (about 901 dollars). Approximately 56.25% of orphans were working in the city, and 18.75% of orphans were farming at country. There was no significant difference in the development status (self-reported health status, monthly income, and current working status) between orphaned youth and the controls (*ps* > 0.05). All subjects were right-handed, had normal or correct-to-normal vision, reported no history of neurological diseases or injury, and had no structural brain abnormality. Before data collection, participants were made aware of the potential risks involved in the study and provided their written informed consent.

**TABLE 1:** Individual characteristics of the sample.

	Overall	Orphans	Controls
***N* (%)**	64 (100%)	32 (50%)	32 (50%)
Male	34 (53.12%)	16 (50%)	18 (56.25%)
Female	30 (46.88%)	16 (50%)	14 (43.75%)
Mean age in years (SD)	25.79 (3.14)	26.65 (2.70)	25.99 (1.97)
**Self-reported health status**			
Very good	50 (78.13%)	25 (78.13%)	25 (78.13%)
Good	11 (17.19%)	5 (15.62%)	6 (18.75%)
Fair	3 (4.68%)	2 (6.25%)	1 (3.12%)
Poor	0 (0%)	0 (0%)	0 (0%)
**Monthly income**			
≤3,000	28 (43.75%)	14 (43.75%)	14 (43.75%)
3,001–6,000	23 (35.94%)	12 (37.50%)	11 (34.38%)
≥6,001	13 (20.31%)	6 (18.75%)	7 (21.87%)
**Current working status**			
Farming at country	10 (15.63%)	6 (18.75%)	4 (12.50%)
Study in college	9 (14.06%)	4 (12.50%)	5 (15.63)
Work in the city	39 (60.94%)	18 (56.25%)	21 (65.62%)
Work for the government	6 (9.37%)	4 (12.50%)	2 (6.25%)

Among these 64 participants, 16 orphans and 16 controls received social exclusion, and 16 orphans and 16 controls received social inclusion. One orphaned youth (male) was excluded from the further analysis due to excessive artifacts. A total of 31 orphaned youth (15 female, mean age = 26.16) and 32 control groups (14 female; mean age = 25.02), matched with age and development status, successfully completed a computerized version of the Trust Game while recording EEG. They engaged in a 2 (group: orphans, controls) × 2 (condition: inclusion, exclusion) between-participants design.

### Stimuli and Procedure

Cyberball was used to induce social exclusion (25, 31, 32), and is the widely used research paradigm in cognitive neuroscience research on social exclusion (33, 34), which needs subjects to participate in an online virtual throwing-the-ball game. Participants, assigned randomly to exclusion and inclusion group, were told to play the game with another two players and to imagine the game situation, which was more important than the performance in the task. The number of the toss of ball is 30. In the exclusion group, participants caught the ball only at the beginning and never again (two tosses). While in the inclusion group, participants were just as likely to catch the ball as the other players (10 tosses). In fact, there were no other players, and the player’s throw is predetermined. Immediately following the Cyberball game, all participants completed a 24-item Needs-Threat Scale, including a 14-item Basic Needs Questionnaire and an 8-item mood measure, along with 2-item, estimated their percentage of game participation and ball receipt (35). All responses had a 9-point Likert-type scale and were reverse-scored where appropriate so that higher numbers indicated more fulfillment of the particular need and a more positive mood.

Then, participants were informed to play a Trust Game with their partners from Cyberball game. In fact, the partner is virtual, during which their EEG activities were recorded. The Trust Game task used in this study is based on the original investment/Trust Game of (36), in which the participants need to act as trustors and complete 150 rounds of games. Before the beginning of each round, both the trustor and the trustee will receive an initial fund of 10 points. The participant (trustor) needs to decide whether to give all 10 points to the trustee. If the participant choose not to give it to the trustee, the current round of the game ends, and both parties receive their own 10-point appearance fee; If the participants choose to give them to the trustee, these points will be tripled to the trustee, and the trustee will decide how to distribute their points (the original 10 points appearance fee plus the doubled 30 points, a total of 40 points). The trustee has two options: sharing all points equally or swallowing all points. No matter which allocation option is selected, the current round of the game ends and both parties get corresponding points.

**FIGURE 1:**
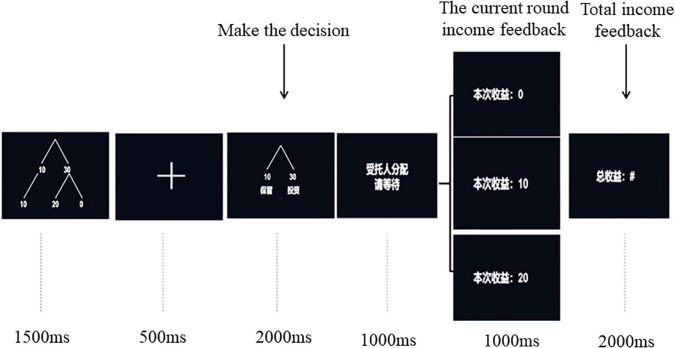
Procedures for the Trust Game.

After finishing the Cyberball game, the experimenter introduced the rules of Trust Game to participants in detail. Then, the participants sat comfortably on the chair in a quiet and sound attenuated electric shielding room, about 1 m away from the computer screen, and began to wear electrode caps and other preparations. The presentation order of stimuli in each round was shown in Figure 1. At the beginning of each round, a sample decision tree of the Trust Game (1,500 ms) was presented to prompt all possible options and results of the current task. After a cross lasting for 500 ms, a decision option diagram (2,000 ms) was presented on the computer screen, and participants needed to press the key to make the decision of trust or distrust within the presentation time of the decision option diagram. Participants were instructed to press “F” when they selected trust and press “J” when they selected distrust. It was regarded as invalid data if it exceeds 2,000 ms. Then, after a prompt waiting screen of 1,000 ms, the results of the current round (1,200 ms) and the total accumulated income (2,000 ms) were presented on the screen.

### Event-Related Potentials Data Recording and Analysis

The EEG was recorded from 32 channels using the 10–20 system (Brain Products, Gilching, Germany) with a bandpass from 0.01 to 100 Hz and a 500 Hz sampling rate. All channels were online-referenced to FCz during recording. Recording impedance for all electrodes was held beneath 10 kΩ.

After data acquisition, EEG data were transferred into the Brain Vision Analyzer 2.0 software (Brain Products, Munich, Germany), which was used to analyze neurophysiologic data. The EEG was offline, re-referenced to the average of the two mastoids, and filtered with a bandpass of 0.1–30 Hz. Epochs were extracted between the 200-ms pre-stimulus and 800-ms post-stimulus interval. For each ERP, activity in the −200 to 0 ms time window prior to feedback presentation served as a baseline. Ocular (blink and saccade) and any other remaining artifacts (muscular, cardiac) were isolated by independent component analysis’ (ICA) algorithm decomposition. In case of doubt, the rejection occurred only if all researchers involved in the data treatment reached an agreement. Eye movement artifacts and trials with EOG artifacts (e.g., a mean EOG voltage exceeding ±80 μV) were automatically rejected.

This study analyzed the ERP components’ FRN and P300 in the stage of feedback presentation. The FRN peaks approximately to 250 ms following feedback presentation (16, 37). Therefore, the mean amplitudes from 200 to 400 ms following feedback presentation were calculated. The P300 component was measured as the mean amplitude between 300 and 550 ms (38). Amplitudes of the FRN and P300 were measured as mean values (14, 15). The electrodes for further analysis were chosen according to ERP topographical distribution and previous studies (39–41). Statistical analyses were conducted at three midline electrodes: Fz, FCz, and Pz.

### Statistical Analysis

The SPSS 24.0 was used to perform a chi-square test to investigate the manipulation check and behavioral difference between the orphan exclusion (OE) group, orphan inclusion (OI) group, control exclusion (CE) group, and control inclusion (CI) group. To test whether participants perceived the social exclusion as expected, a multivariate analysis of variance (MANOVA) was conducted using the factors of the group (orphans, controls) and condition (inclusion, exclusion). The four ratings of the manipulation check (“basic needs”, “mood”, “percentage of game participation”, and “percentage of ball receipt”) served as dependent variables. A repeated-measures ANOVA was conducted on ERP data with electrode point (Fz, FCz, and Pz) and feedback (gain, loss) as within-subject factors, group (orphans, controls), and condition (inclusion, exclusion) as between-subject factors. For all the analyses in this study, *p* < 0.05 was considered to be statistically significant, and the *p*-values were adjusted using the Greenhouse-Geisser correction when appropriate.

## Results

### Manipulation Check

As shown in Table 2, there was no significant difference between orphans and controls in the four ratings of the manipulation check. The group × condition interaction was significant only for “percentage of ball receipt”, with a lower percentage rating in exclusion compared to the inclusion only for controls. However, both orphans and controls in the inclusion condition reported significantly lower scores in basic needs and mood and had significantly higher rates of game participation and ball receipt than youth in the exclusion condition. In short, social exclusion was induced successfully.

**TABLE 2:** Manipulation check of social exclusion.

Dependent variable	Orphans *n* = 31		Controls *n* = 32		Statistics (*df* = 1, 59)		
			
	Mean	S.D.	Mean	S.D.	Condition	Group	Interaction
**Basic needs** ^a^							
Inclusion	3.53	1.30	3.58	1.02	*F* = 18.21, *p* < 0.001, η*_*p*_*^2^ = 0.24	N.S.	N.S.
Exclusion	5.15	1.54	4.89	1.53			
**Mood** ^a^							
Inclusion	3.09	1.91	2.68	1.17	*F* = 9.89, *p* = 0.003, η*_*p*_*^2^ = 0.14	N.S.	N.S.
Exclusion	4.15	1.26	3.43	1.77			
**Percentage of game participation** ^a^							
Inclusion	7.00	2.07	6.50	1.67	*F* = 39.12, *p* < 0.001, η*_*p*_*^2^ = 0.40	N.S.	N.S.
Exclusion	3.33	1.99	3.75	2.35			
**Percentage of ball receipt** ^a^							
Inclusion	4.96	1.53	6.13	2.03	*F* = 30.32, *p* < 0.001, η*_*p*_*^2^ = 0.34	N.S.	*F* = 5.19, *p* = 0.026, η*_*p*_*^2^ = 0.08
Exclusion	3.53	2.64	2.44	0.96			

*N.S., not significant; condition = inclusion/exclusion, Group = orphans/controls, Interaction = Situation × Group. ^a^Manipulation check ratings from the Needs-Threat Scale.*

### Behavioral Performance

**FIGURE 2:**
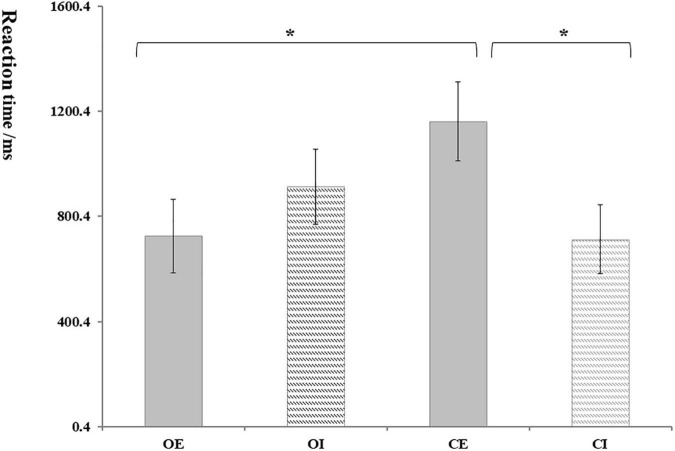
The rate of trust. OE, orphans and exclusion group; OI, orphans and inclusion group; CE, controls and exclusion group; CI, controls and inclusion group. Error bars represent the standard errors. *p < 0.05.

Overall, the trust rate of participants was significantly higher than the distrust rate. In orphans, the rates of trust in the OE group and OI group were 75.50% (SD: 14. 19%) and 64.20% (SD: 17.31%), respectively. In controls, the rates of trust were 57.72% (SD: 13.15%) in the CE group and 55.75% (SD: 13.97%) in the CI group. Multiple comparisons showed that the trust rate of the orphans group was significantly higher than that of the control group (*p* = 0.003), and the trust rate of the OE group was significantly higher than that of the OI group (Figure 2).

**FIGURE 3:**
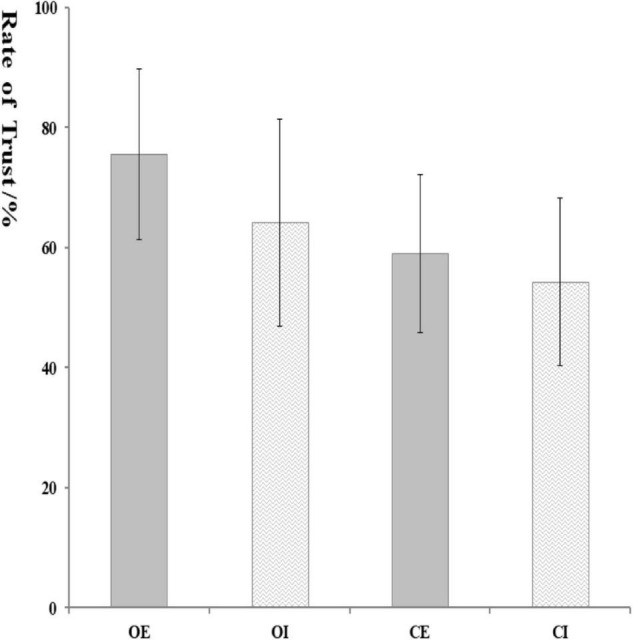
The reaction time of trust decision. Error bars represent the standard errors.

Taking the trust and distrust mean response time of participants as the dependent variables; repeated measure ANOVA results showed that the interaction between group and condition was significant, *F*_(1, 62)_ = 5.17, *p* = 0.027, η*_*p*_*^2^ = 0.09. As shown in Figure 3, the reaction time of the OE group (724.66 ± 139.69) was significantly shorter than that in the CE group (1,161.25 ± 150.06); and the reaction time of the CE group (1,161.25 ± 150.06) was significantly longer than that in the CI group (713.03 ± 131.22).

### Event-Related Potentials Data Analysis

#### FRN

**FIGURE 4:**
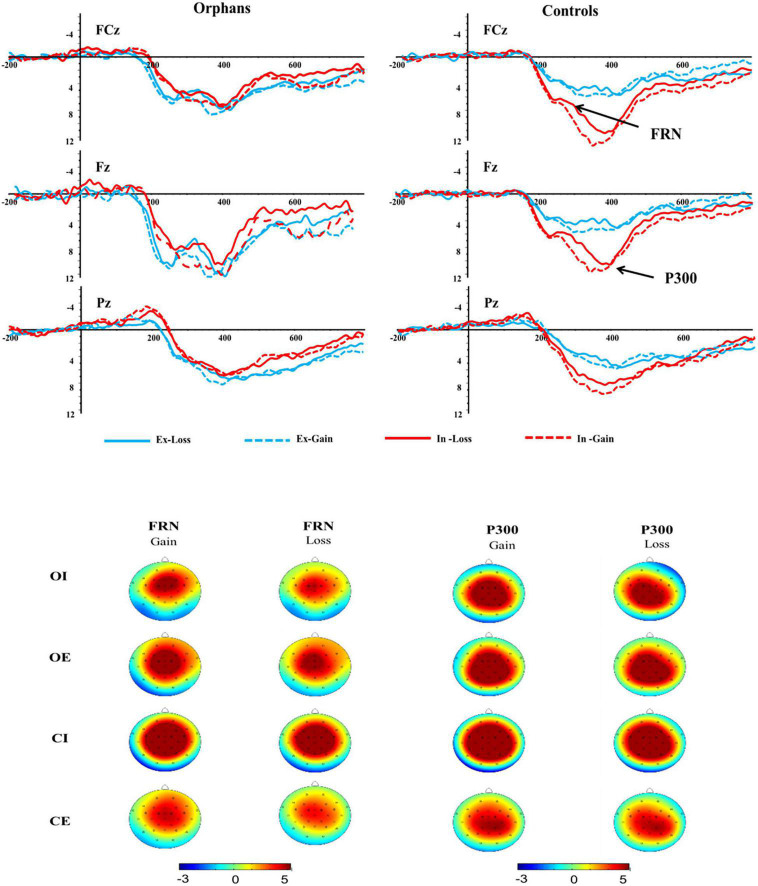
Feedback-related negativity (FRN) and P300 components at FCz, Fz, and Pz electrodes and topographic maps of FRN and P300 scalp voltage.

There was a significant main effect of feedback, *F*_(1, 62)_ = 13.88, *p* = 0.001, η*_*p*_^2^* = 0.19, with loss eliciting more negative FRN (5.01 ± 0.39 μV) than gain (5.75 ± 0.46 μV). The main effect of the electrode was significant, *F*_(1, 62)_ = 13.28, *p* = 0.001, η*_*p*_^2^* = 0.18, for which pairwise comparisons suggested that the FRN amplitude was greater at FCz (6.12 ± 0.50 μV) than at Fz (5.64 ± 0.42 μV) and Pz (4.36 ± 0.37 μV). Results also showed a marginally significant difference in group, *F*_(1, 62)_ = 3.02, *p* = 0.081, η*_*p*_^2^* = 0.49, with orphans eliciting more negative FRN (4.65 ± 0.60 μV) than controls (6.10 ± 0.59 μV), see Figure 4.

**FIGURE 5:**
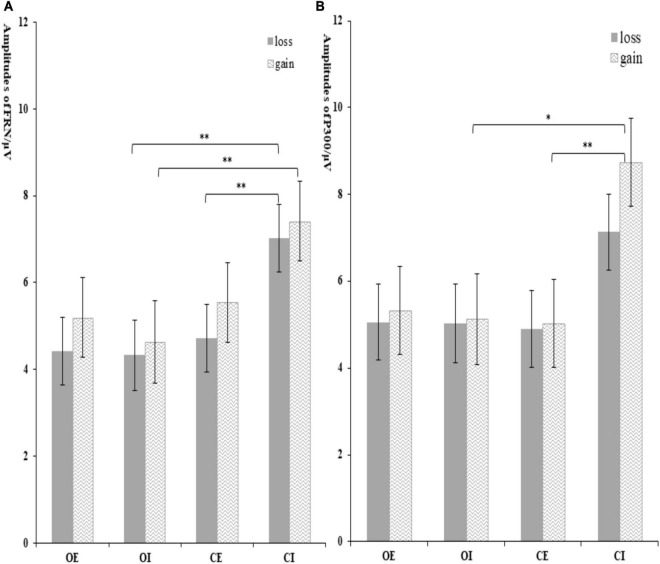
The amplitude of FRN **(A)** and P300 **(B)** at different conditions. Error bars represent the standard errors. *p < 0.05 and **p < 0.01.

The interaction effect of group × condition was significant, *F*_(1, 62)_ = 5.03, *p* = 0.029, η*_*p*_^2^* = 0.80. Further analysis indicated that the significant group difference was only found in inclusion condition, with a more negative FRN in orphans (4.38 ± 0.83 μV) compared to controls (7.82 ± 0.83 μV), but not in exclusion. The interaction effect of group × condition × feedback was significant, *F*_(1, 62)_ = 4.60, *p* = 0.036, η*_*p*_^2^* = 0.08. As shown in Figure 5A, the significant group difference was only found in inclusion and loss, with a more negative FRN in orphans (4.33 ± 0.81 μV) compared to controls (7.02 ± 0.78 μV). From another perspective, the CE group (4.22 ± 0.78 μV) elicited a more negative FRN than CI group (7.02 ± 0.78 μV) only in loss, while there was no significant difference between the OE and OI groups.

#### P300

The P300 amplitudes were entered into a 2 (group) × 2 (condition) × 2 (feedback) × 3 (electrode) ANOVA (see Figure 4). The main effect of feedback was significant, *F*_(1, 62)_ = 3.79, *p* = 0.056, η*_*p*_*^2^ = 0.06, with gain (5.98 ± 0.51 μV) eliciting larger P300 than loss (5.53 ± 0.44 μV). There was also a significant main effect of electrode, *F*_(1, 62)_ = 34.28, *p* < 0.001, η*_*p*_*^2^ = 0.34, with the P300 amplitude being greater at FCz (6.22 ± 0.53 μV) than at Pz (5.56 ± 0.47 μV) and Fz (5.48 ± 0.45 μV).

The interaction effect of group × condition was marginally significant, *F*_(1, 62)_ = 3.05, *p* = 0.082, η*_*p*_*^2^ = 0.50. Further analysis indicated that the significant group difference was only found in inclusion condition, with a larger P300 in controls (7.93 ± 0.92 μV) compared to orphans (5.08 ± 0.95 μV), but not in exclusion. The CI group (7.93 ± 0.92 μV) elicited a larger P300 than that in CE group (4.81 ± 0.92 μV). The significant feedback × group × condition interaction effect indicated that the significant group difference was only found in gain and inclusion condition, *F*_(3, 60)_ = 2.95, *p* = 0.04, η*_*p*_^2^* = 0.13, with an overall attenuated P300 in orphans (5.13 ± 1.04 μV) compared to controls (8.73 ± 1.01 μV), but not in exclusion condition (see Figure 5B).

## Discussion

Most of the previous studies about the influence of social exclusion on trust were conducted in the form of questionnaires, and there was a lack of experimental methods to investigate the trust performance after experiencing social exclusion. Furthermore, orphaned youth were invited to participate in this study to explore the influence of social exclusion on trust and the neurophysiological mechanism, which had important practical significance for the social adaptation of orphaned youth.

Behavioral task results showed that the trust ratio of the OE group was significantly higher than that of other groups, and the response time of the OE group was the shortest. Although these results are not consistent with our hypothesis, it clearly suggests that orphaned youth preferred to believe others when they encountered social exclusion. This finding is consistent with Williams et al. (32), who found that the social exclusion induced by the Cyberball game did lead to some verbal or active behaviors to get others’ attention and response. The possible reason for this result is when orphaned youth are in a vulnerable position in interpersonal communication, they often choose to show kindness to others to get a response. Moreover, this high rate of trust in orphaned youth may be related to the risk decision-making after social exclusion (42). Studies have found that excluded individuals have more risk-taking behaviors (43) and higher risk preference (44), showing risk-seeking in risk decision-making tasks (45). In the Trust Game of this study, choosing trust represents the risky behavior of investment.

The ERP results showed that the FRN amplitude of orphans (OI) was significantly greater than that of controls (CI) only in inclusion condition and loss feedback. Consistent with prior research on the effect of FRN, the result we found indicated that FRN is very sensitive to the valence of results, reflecting the processing of negative results in the anterior cingulate cortex (22, 40). The rational choice theory holds that FRN is responsible for coding the deviation between feedback results and prior expectations. In our study, the OI group chose to trust others, indicating that they were willing to cooperate with the other party for a win-win, and subjectively expected the other party to choose reciprocity. Therefore, the loss feedback would cause a greater expected decision than the gain feedback, thus, inducing a larger FRN amplitude. Similar to the results of this study, Long et al. (46) found that trust could modulate the amplitude of FRN, compared with the subjects who chose distrust, the amplitude of FRN induced by the subjects who chose trust was larger. Furthermore, researchers pointed out that the loss of FRN effect under the condition of exclusion might be caused by a sense of aloofness, which made participants less likely to anticipate the subsequent results (17). In short, social exclusion may decrease orphaned youths’ expectations of reciprocity, resulting in a sense of aloofness and lower expectations of the results of others’ feedback. To some extent, this reflects that orphaned youth are more likely to be accepted by others in society and they are more sensitive to others’ feedback when they are accepted. The current data suggested that in a social context, social exclusion tendencies affect how outcomes were evaluated by orphaned youth. It also provided support that FRN is sensitive to the affective properties of social pain.

Interestingly, although we found that gain induced a larger P300 amplitude in the OI group than loss, there was no significant group difference in P300 between the OI group and the OE group. It is generally believed that P300 is associated with the allocation of attention resources in outcome evaluation and a high level of motivation/emotion evaluation (13, 37). These results suggested that social exclusion did not affect the orphaned youths’ motivational/emotional evaluations of outcome feedback. We tried to explain this result, according to the emotional numbness theory proposed by Baumeister et al. (47), we suspected that the early negative experiences could cause orphaned youth to become numb, including emotional and physical numbness. This defensive response of self-protection temporarily could reduce the pain and enable them to cope with the negative events. This point can be demonstrated by our results that the P300 amplitude was larger in CI group than OI group. Unlike orphaned youth, the amplitude of P300 induced by CI group was larger than that by CE group, which indicated that social exclusion significantly reduced the sensitivity and the level of motivational/emotional evaluation of the feedback results in control group. The P300 component reflects the late resource allocation in the Trust Game. When the participants encountered social exclusion, they might consume a lot of resources, leading to a sharp decrease in subsequent resources, and attracting less attention in the outcome evaluation. The CI group had a higher level of motivational/emotional meaning due to an inclusive environment, leading to the larger P300 amplitude. The study of Rigoni et al. (48) also found that complex social situations would weaken individual’s attention to winning or losing results.

Our study has some limitations. Firstly, although we controlled some key contextual and individual factors, such as gender and age, data on some key contextual and individual factors, such as current social status, were not available in the current study. Future studies need to take into consideration of these factors to get a better understanding of the effect of social exclusion on orphaned youths’ trust. Secondly, as this study was conducted in China, the generalizability of the finding to other settings may be limited. Comparing effects across culture or social value orientations would supplement this study to provide a more comprehensive understanding of decision-making after social exclusion. Finally, another limitation pertains to the sample size. Our sample size may be relatively small due to the particularity of the subjects and the increasing difficulty of following up after 15 years. Future studies should further increase the sample size to improve the validity of the study.

## Conclusion

The current study attempts to demonstrate the behavioral performance and neurophysiological mechanism of trust in orphaned youth with existing stigma experience when they encounter social exclusion in the laboratory. The results found that orphaned youth had contradictory and complex psychological responses. On the one hand, in terms of behavioral responses stage, orphaned youth showed significantly more trusting behaviors; on the other hand, in terms of outcome evaluation stage, orphaned youth showed sensitivity to the deviation of feedback and lower motivational/emotional evaluations of reward. To a certain extent, these findings indicated that orphaned youth might have formed some type of self-protective mechanism to prevent the negative feedback of others from causing a serious blow to themselves. In other words, their previous negative experiences may also play an important role, so we suggest that people should reduce the stigma and exclusion behavior of vulnerable groups, such as orphaned youth, and give them more positive feedback, which may contribute to their interpersonal social adaptation. Simultaneously, it has important implications for understanding the processes by which social exclusion may adversely affect the mental health of youth orphaned by HIV/AIDS.

## Data Availability Statement

The datasets presented in this article are not readily available because of privacy or ethical restrictions. Requests to access the datasets should be directed to JZ, jfzhao63@hotmail.com.

## Ethics Statement

The studies involving human participants were reviewed and approved by Institutional Review Board at the Henan University in China (IRB 00007212). The patients/participants provided their written informed consent to participate in this study.

## Author Contributions

JW, QZ, and JZ designed the study and drafted the manuscript. YZ and LJ performed the study. JW, QZ, and SQ analyzed the data and edited the manuscript. JZ and XL revised the manuscript. All authors contributed to the article and approved the submitted version.

## Conflict of Interest

The authors declare that the research was conducted in the absence of any commercial or financial relationships that could be construed as a potential conflict of interest.

## Publisher’s Note

All claims expressed in this article are solely those of the authors and do not necessarily represent those of their affiliated organizations, or those of the publisher, the editors and the reviewers. Any product that may be evaluated in this article, or claim that may be made by its manufacturer, is not guaranteed or endorsed by the publisher.
